# Plant Polysaccharide*s* Modulate Biofilm Formation and Insecticidal Activities of *Bacillus thuringiensis* Strains

**DOI:** 10.3389/fmicb.2021.676146

**Published:** 2021-06-28

**Authors:** Mengmeng Li, Changlong Shu, Wang Ke, Xiaoxiao Li, Yiyan Yu, Xiong Guan, Tianpei Huang

**Affiliations:** ^1^State Key Laboratory of Ecological Pest Control for Fujian and Taiwan Crops & Key Laboratory of Biopesticide and Chemical Biology of Ministry of Education & Ministerial and Provincial Joint Innovation Centre for Safety Production of Cross-Strait Crops, College of Life Sciences & College of Plant Protection, Fujian Agriculture and Forestry University, Fuzhou, China; ^2^State Key Laboratory of Plant Diseases and Insect Pests Biology, Institute of Plant Protection, Chinese Academy of Agricultural Sciences, Beijing, China

**Keywords:** *Bacillus thuringiensis*, biofilm, pectin, insecticidal activity, xylan, *Plutella xylostella*, plant polysaccharide

## Abstract

After the biological pesticide *Bacillus thuringiensis* (Bt) is applied to the field, it has to remain on the surface of plants to have the insecticidal activities against insect pests. Bt can form biofilms on the surface of vegetable leaves, which were rich in polysaccharides. However, the relationship between polysaccharides of the leaves and the biofilm formation as well as the insecticidal activities of Bt is still unknown. Herein, this study focused on the effects of plant polysaccharides pectin and xylan on biofilm formation and the insecticidal activities of Bt strains. By adding pectin, there were 88 Bt strains with strong biofilm formation, 69 strains with weak biofilm formation, and 13 strains without biofilm formation. When xylan was added, 13 Bt strains formed strong biofilms, 98 strains formed weak biofilms, and 59 strains did not form biofilms. This indicated that two plant polysaccharides, especially pectin, modulate the biofilm formation of Bt strains. The ability of pectin to induce biofilm formation was not related to Bt serotypes. Pectin promoted the biofilms formed by Bt cells in the logarithmic growth phase and lysis phase at the air–liquid interface, while it inhibited the biofilms formed by Bt cells in the sporangial phase at the air–liquid interface. The dosage of pectin was positively correlated with the yield of biofilms formed by Bt cells in the logarithmic growth phase or lysis phase at the solid–liquid interfaces. Pectin did not change the free-living growth and the cell motility of Bt strains. Pectin can improve the biocontrol activities of the spore–insecticidal crystal protein mixture of Bt and BtK commercial insecticides, as well as the biofilms formed by the logarithmic growth phase or lysis phase of Bt cells. Our findings confirmed that plant polysaccharides modulate biofilm formation and insecticidal activities of Bt strains and built a foundation for the construction of biofilm-type Bt biopesticides.

## Introduction

Biofilms are abundantly present in insect intestines, phyllospheres, rhizospheres, and soils. Bacterial biofilms are aggregates of bacteria embedded in extracellular polymeric substances (EPSs) produced by the bacteria themselves. Their EPSs mainly contain extracellular polysaccharides, proteins, lipids, and extracellular DNA, which can adhere to interfaces of solid–liquid, solid–gas, liquid–liquid, and liquid–gas (Flemming et al., 2016; Flemming and Wuertz, 2019). In fact, bacteria can colonize almost all kinds of natural or synthetic surfaces and form biofilms (Hall-Stoodley et al., 2004; Sweet et al., 2011; Flemming and Wuertz, 2019). Approximately 80% of the bacteria and archaea on the earth exist in the form of biofilms. The average numbers of bacteria in the biofilms locating at termite intestines, phyllospheres, and soil on the land surface are 6 × 10^23^, 2 × 10^26^, and 3 × 10^29^, and the average number of bacteria in the rhizosphere biofilms may be comparable to that of the rhizospheres (Flemming and Wuertz, 2019).

Compared with planktonic cells, the most important feature of bacterial biofilm is that it can help bacteria resist different environmental stresses such as ultraviolet (UV) radiation, antibiotic treatment, drying, temperature variation, oxidative stress, nutrient depletion, and pH change (Rinaudi and Giordano, 2010; Bernbom et al., 2011; Koerdt et al., 2011; Dang and Lovell, 2016).

Plant-derived substances are related to the formation of bacterial biofilms. Natural phenolic compounds have a significant inhibitory effect on the formation of *Pseudomonas aeruginosa* biofilm (Jagani et al., 2009). Laminaria polysaccharides, xylan, and k-carrageenan can promote the biofilm formation of *Xylella fastidiosa in vitro*, whereas lichen polysaccharides, oligosaccharides, k-carrageenan, laminarin, xylan, and β-D-glucan can significantly reduce its cell aggregation (Cheng et al., 2009). The fungal supernatants induced by arabinose and pectin can effectively remove the biofilm of *Pseudomonas fluorescens* (Orgaz et al., 2007). Pectin increased the cell viscosity of *X. fastidiosa* (Killiny and Almeida, 2009). Plant polysaccharides (xylan, pectin, arabinose) can stimulate the formation of *Bacillus subtilis* biofilm, which can be used to regulate the phosphorylation state of the main regulator Spo0A. In addition, plant polysaccharides can also be used to synthesize the carbon of the EPSs (Beauregard et al., 2013).

*Bacillus thuringiensis* (Bt) can produce proteinaceous crystalline inclusions (crystals) during sporulation, which are responsible for its toxicity toward a variety of invertebrates, especially insects (Sauka and Benintende, 2008). Its crystal proteins can be divided into different categories based on amino acid similarity. However, some Bt strains contain a complex family of *cry* genes. For example, HD1 expressed Cry1Aa, Cry1Ab, Cry1Ac, Cry1Ia, Cry2Aa, and Cry2Ab proteins. But HD73 had only one Cry protein (Cry1Ac) (Sauka et al., 2010). Bt is a widely used agricultural and forestry microbial pesticide (Zwick et al., 2012; Huang et al., 2016, 2018). As early as 2016, the global biocontrol market, including Bt and virus products, exceeded $1 billion^1^. In addition, HD1 and HD73 have been applied to produce mainstream Bt commercial insecticides. In 1969, HD1 was isolated by Dulmage HT, and its insecticidal ability was 20–200 times that of other Bt strains. In the following years, American manufacturers turned to produce HD1 preparations, becoming the main products of agriculture and forestry control. Hence, HD1 greatly promoted the commercialization of BtK insecticides. HD73 strain, isolated by Dulmage HT, was also extensively applied in Bt industry (Krywienczyk et al., 1978). The main active ingredients of Bt products are spores and insecticidal crystal proteins (ICPs). During the application process, Bt vegetative cells, spores, and their ICPs are sensitive to UV radiation, resulting in a relative short duration of Bt under field conditions. Researchers had tried UV radiation, addition of protective agents, construction of melanin gene *mel* genetic engineering bacteria, gene knockout of Bt mother cell lysis gene *cwlC*, and microcapsules to improve Bt’s UV resistance and thus enhance its toxicity to insect pests (Ruan et al., 2005; Chen et al., 2016; He et al., 2017; Zhang et al., 2018). Unfortunately, because of practicability, cost, difficulty in the approval of genetic engineering microbial pesticides, and docking of industry, academia, and research, these technologies are rarely used in Bt industrial production and field application. Herein, there is an urgent need to propose practical, high-quality, and inexpensive solutions to develop new Bt preparations with better insecticidal effects.

Bt can be colonized on the surface of cotton, cabbage, clover, and peas. The colonization time of Bt on the surface of cotton can be as long as 8 weeks, and the colonized leaves have certain insecticidal activity against *Plutella xylostella* and *Spodoptera frugiperda* (Normander et al., 1998; Bizzarri and Bishop, 2007, 2008; Bizzarri et al., 2008; Monnerat et al., 2009). *P. xylostella* (Lepidoptera: Plutellidae), the major insect pest of *Brassica* vegetable crops in southern China, occurs yearly and causes heavy damage (Wei et al., 2013). Because of the frequent use of biopesticides, *P. xylostella* is resistant to Bt preparations in some areas. The resistance of *P. xylostella* to Bt preparations was first discovered in a field trial in Hawaii. However, *P. xylostella* had low resistance to Bt or no resistance in most areas. A recent study had surveyed six representative populations of *P. xylostella* collected in Shanghai, Shandong, Hubei, Hunan, Zhejiang, Guangdong, and other places in China. The bioassay results showed that *P. xylostella* populations from different locations showed different resistance levels. The resistance of Guangdong field population to BtK preparation was 21.90 times. The populations of Shanghai, Hunan, Shandong, and Zhejiang had certain resistance to Cry1Ac, but the resistance to BtK preparation was not obvious. The Hubei population showed no resistance to Cry1Ac and BtK (Wang et al., 2007). However, the effects of plant polysaccharides on the formation and insecticidal activity of Bt biofilms are still unknown. Therefore, this article focuses on the effects of plant polysaccharides, especially pectin, on the regulation of the biofilm formation and insecticidal activity of Bt to build a foundation for the construction of biofilm-type Bt biopesticides.

## Materials and Methods

### Bt Strains

Most of Bt strains used in this study were obtained from the Bacillus Genetic Stock Center of Ohio State University as follows: 4A1, 4A2, 4A3, 4A4, 4A5, 4A6, 4A7, 4A8, 4A9, 4A10, 4A11T, 4B1, 4B2, 4B3, 4B4, 4B5, 4C1, 4C2, 4C3, 4D1, 4D2, 4D3, 4D4, 4D5, 4D12, 4D16, 4D17, 4D18, 4D19, 4E2, 4E6, 4E7, 4F1, 4F2, 4F3, 4F4, 4F5, 4G1, 4G2, 4G3, 4G4, 4G5, 4G6, 4G7, 4G8, 4H1, 4H2, 4H3, 4H4, 4H5, 4I1, 4I2, 4I3, 4I4, 4I5, 4I6, 4J1, 4J2, 4J3, 4J4, 4J5, 4J6, 4J7, 4J8, 4K1, 4K3, 4K5, 4K6, 4K7, 4K8, 4K9, 4L1, 4L2, 4L3, 4L4, 4L5, 4L6, 4M1, 4M2, 4M3, 4M4, 4M5, 4M6, 4N1, 4O1, 4P1, 4P2, 4Q1, 4Q3, 4Q4, 4Q12, 4R1, 4S2, 4S3, 4T1, 4U1, 4V1, 4V3, 4V6, 4W1, 4W2, 4X1, 4Y1, 4Z1, 4AA1, 4AC1, 4AE1, 4AF1, 4AG1, 4AH1, 4AK1, 4AL1, 4AM1, 4AN1, 4AO1, 4AP1, 4AQ1, 4AR1, 4AS1, 4AT1, 4AU1, 4AV1, 4AW1, 4AX1, 4AY1, 4AZ1, 4AZ2, 4AZ3, 4BA1, 4BB1, 4BC1, 4BD1, 4BE1, 4BF1, 4BG1, 4BH1, 4BJ1, 4BK1, 4BL1, 4BM1, 4BN1, 4BP1, 4BQ1, 4BR1, 4BS1, 4BT1, 4BU1, 4BV1, 4BW1, 4BX1, 4BY1, 4BZ1, 4CA1, 4CB1, 4CC1, 4CD1, 4CE1, 4CG1, 4XX1, 4XX2, 4XX3, 4XX4, and 4XX5. The rest of Bt strains deposited in the biotechnology group of the Institute of Plant Protection, Chinese Academy of Agricultural Sciences, China, were as follows: 261–1, JJX, G03, HD1, Bt185, 1126–1, HBF-18, HD73, G033A, and KN11.

### Effects of Plant Polysaccharides Pectin and Xylan on the Biofilm Formation of Bt

MSN medium (5 mM K_3_PO_4_⋅3H_2_O, 100 mM MOPS, 50 μM MnCl_2_⋅4H_2_O, 700 μM CaCl_2_, 2 μM thiamine, 1 μM ZnCl_2_, 2 mM MgCl_2_⋅6H_2_O, 0.2% NH_4_Cl, pH 7.0) without plant polysaccharides pectin or xylan was used as the control group. One hundred milliliters of MSN medium were mixed with 50 mL of 15 mg/mL pectin or xylan. One milliliter of MSN medium with or without pectin or xylan was added to each well in the 24-well culture plates. Then, 13.5 μL of an overnight culture of Bt strains with OD_600_ ≈ 0.3 was added into each well and incubated at 30°C for 48 h. In this experiment, the method of qualitative analysis was used to analyze the effect of pectin on the formation of biofilm by Bt strains after cultured in a 24-well plate (Beauregard et al., 2013).

In order to explore whether the ability of pectin to induce biofilms was related to the serotypes of the Bt strains, the difference of biofilm formation induced by pectin in the Bt serotypes with more than five strains was analyzed.

### Effect of Plant Polysaccharide Pectin on the Biofilms Formed by Bt Cells in Different Growth Phases at Solid–Liquid Interfaces

The biofilm induction effect of plant polysaccharide pectin was significantly higher than that of plant polysaccharide xylan. Therefore, the follow-up researches were focused only on plant polysaccharide pectin. Bt strains HD1, HD73, Bt185, HBF18, Bt185, G033A, and KN11 that are currently used for industrial production were selected to test the effect of pectin on the biofilm formation of Bt logarithmic growth phase cells. Briefly, the overnight cultured Bt strains were incubated to their logarithmic growth phases. Then, they were 1% transferred to 200 mL LB liquid medium (10 g/L tryptone, 5 g/L yeast extract, 10 g/L NaCl, pH 7.4) with or without 5 mg/mL pectin in a 250-mL flask and incubated at 30°C for 72 h. Then, the biofilms were harvested, dried, and weighed for quantitative analysis. The Bt strains that can form stronger biofilms induced by pectin were selected to further test the influence of pectin in the biofilm formation of Bt sporangial phase cells.

The above method was referred to in order to determine the effect of pectin on the biofilm formation of Bt spore–ICP mixture. Briefly, the mixture was prepared by liquid fermentation. The overnight cultured Bt strains were 2% transferred to 50 mL 1/2 LB liquid medium in a 250-mL flask containing and cultured with shaking at 220 revolutions/min (rpm) at 30°C until 50% of the spores were released. The Bt fermentation broth was centrifuged at 5,000 rpm for 5 min and washed three times with 0.5 M NaCl and sterile ddH_2_O. And the pellets were diluted with LB medium to 10^8^ colony-forming units (CFUs)/mL to prepare the Bt spore–ICP mixture.

### Effect of the Dosage of Plant Polysaccharide Pectin on the Biofilm Formed by Bt Cells in Different Growth Phases at Solid–Liquid Interfaces

Overnight cultured Bt strains were 1% transferred to LB medium and cultured at 30°C with shaking at 220 rpm to an OD_600_ ≈ 1.00. Then, the culture was 1% transferred to LB medium with 20, 10, 5, 2.5, 1.25, 0.625, and 0 mg/mL pectin. Two milliliters of the mixture was transferred into each well of 24-well plates. After sealing, the plates were treated at 30°C in a moist environment. After 72 h, the OD_600_ values of each well were determined in a microplate reader. Then, a pipette was used to carefully discard the culture medium in the wells, which were rinsed with 2 mL ddH_2_O three times to remove the non-adherent planktonic bacteria in the wells. The wells were allowed for air to dry at room temperature and then stained with 2 mL 0.1% crystal violet for 20 min. The wells were rinsed with 2 mL ddH_2_O three times. After the 24-well plates were air-dried, the dye was dissolved in the wells with 2 mL of absolute ethanol, and the plates were placed on a low-speed shaker to shake for 20 min. Then, the OD_595_ value of each well was measured with a microplate reader to quantify the biofilm formation.

### Effect of Plant Polysaccharide Pectin on the Growth of Bt

Overnight culture of Bt strain was 1% transferred to LB medium with shaking at 220 rpm at 30°C until the OD_600_ was ∼1.00. The culture was 1% transferred to 200 mL LB medium with or without 5 mg/mL pectin in a 250-mL flask and cultured at 30°C with shaking at 220 rpm. The OD_600_ values were measured with a spectrophotometer (Cuevas and Edwards, 2017).

### Effects of Plant Polysaccharide Pectin on the Cell Motility of Bt

The cell motility was tested by the semisolid plate method (Gnasekaran and Subramaniam, 2015). Overnight cultures of Bt strains were 1% transferred to LB medium and cultured at 30°C with shaking at 220 rpm until the OD_600_ values were ∼1.00. One microliter of the Bt culture was mixed with 1 μL of LB medium and 1 μL 10 mg/mL pectin (or LB medium). Then, 2 μL mixture was dropped onto a 5-mm round filter paper in the center of a 9-cm LB plate with 0.3, 0.5, and 0.7% agar. After incubation at 30°C for 24 h, the diameter of the culture was measured.

### Effect of Plant Polysaccharide Pectin on the Toxicities of Spore–ICP Mixtures of Bt Against *P. xylostella*

The spore–crystal mixtures of Bt HD1 and HD73 were prepared according to the method described in the section *Effect of Plant Polysaccharide Pectin on the Biofilms Formed by Bt Cells in Different Growth Phases at Solid–Liquid Interfaces*. The mixtures were resuspended in sterile ddH_2_O, and their spore numbers were determined by the viable counting. The mixtures were diluted with sterile ddH_2_O to prepare 1.7 × 10^13^ and 0.85 × 10^13^ CFU/mL of Bt HD1, and 2.65 × 10^15^ and 1.325 × 10^15^ CFU/mL of Bt HD73. Then, 0, 5, and 25 mg/mL of pectin were added to them. The bioassay of *P. xylostella* of the spore–ICP mixtures was performed as follows: uniformly sized leaves that were not sprayed with pesticides were rinsed with water or the spore-ICP mixtures for 10 s and dried in the air; the leaves were soaked for 5 min in the mixtures with 0.1% detergent and air-dried. Then, the leaves were placed in 90-mm plates with absorbent papers; 30 3rd-instar larvae were transferred to each treatment, and the plates were strictly sealed with two-layer absorbent papers; after incubation in a light incubator for 2 days at 25°C, the number of dead and alive larvae was used for the calculation of corrected mortality.

### Effect of Plant Polysaccharide Pectin on the Toxicity of the Cry1Ac Protein of Bt Against *P. xylostella*

In order to explore whether the promotion of plant polysaccharide pectin on the bioassay of the spore–ICP mixture of Bt HD73 with one ICP (Cry1Ac) against *P. xylostella* was related to Cry1Ac protein, the effect of 25 mg/mL pectin on the toxicity of the Cry1Ac extracted from Bt HD73 against *P. xylostella* was investigated.

Overnight cultured Bt HD73 was 1% transferred to a 1-L flask containing 300 mL of 1/2 LB medium and incubated at 30°C with shaking at 220 rpm. When 50% of spores were released, the fermentation product was centrifuged at 8,000 rpm, 4°C, for 15 min. Then, the pellets were washed with 1 M NaCl and centrifuged at 9,000 rpm, 4°C, for 15 min. After washed with sterile ddH_2_O, 3% β-mercaptoethanol was used to resuspend the pellets (50 mL β-mercaptoethanol/1-L culture), pH ∼9.5–10. After the samples were placed on ice with shaking at 110 rpm for 4–10 h, they were centrifuged at 8,000 rpm and 4°C for 15 min. Then, the supernatants were mixed with 4 M NaAc-HAc (pH 4.5) in a ratio of 7:1, and the pH values were adjusted to ∼4.5–5.0. After 4 h at 4°C, the samples were centrifuged at 8,000 rpm, 4°C, for 15 min, and the pellets were washed three times with precooled sterile ddH_2_O. Then, the pellets, Bt HD73 Cry1Ac protein, were completely dissolved in 50 mM Na_2_CO_3_ (pH 9.5) with a stir.

Sodium dodecyl sulfate–polyacrylamide gel electrophoresis (Hofte and Whiteley, 1989; Laemmli, 1970) was used to detect the Cry1Ac protein. After filtration with a 0.22-μm filter membrane, the protein was quantified with the Bradford method; 100, 33.33, 11.11, 3.703, and 1.345 μg/mL of Cry1Ac protein was prepared with phosphate-buffered saline buffer for bioassay. The treatment group was added with 25 mg/mL pectin. The bioassay of *P. xylostella* with Bt HD73 Cry1Ac was carried out as the aforementioned method.

### Effect of Plant Polysaccharide Pectin on the Toxicity of Commercial BtK Preparation Against *P. xylostella*

Commercial BtK preparation 1.6 × 10^2^ and 1.6 × 10^3^ IU/mL with and without 25 mg/mL pectin was prepared. *P. xylostella* bioassay was carried out according to the above method, and the LC_50_ values, which cause 50% corrected mortality of the insect pest, were calculated with or without the induction of 25 mg/mL pectin.

### Effect of Plant Polysaccharide Pectin on the Toxicities of Bt Biofilms Against *P. xylostella*

The biofilms formed by Bt cells in the logarithmic growth phase, sporangial phase, and lysis phase were prepared according to the method described in the section of *Effect of Plant Polysaccharide Pectin on the Biofilms Formed by Bt Cells in Different Growth Phases at Solid–Liquid Interfaces*. Ten milliliters of sterile ddH_2_O was added to each biofilm sample. The treatment group was added with 25 mg/mL pectin. *P. xylostella* bioassays of Bt biofilms were carried out with reference to the aforementioned method.

### Statistical Analysis

The above treatment was set up with three replicates of three samples. Analysis of variance with Duncan test of the differences between groups was carried out using SPSS 25.0. And the data were expressed as mean ± standard deviation. *P* < 0.05 and *P* < 0.01 represented statistically significant differences and statistically extremely significant differences, respectively.

## Results

### Plant Polysaccharides Modulate the Biofilm Formation of Bt Strains

**FIGURE 1:**
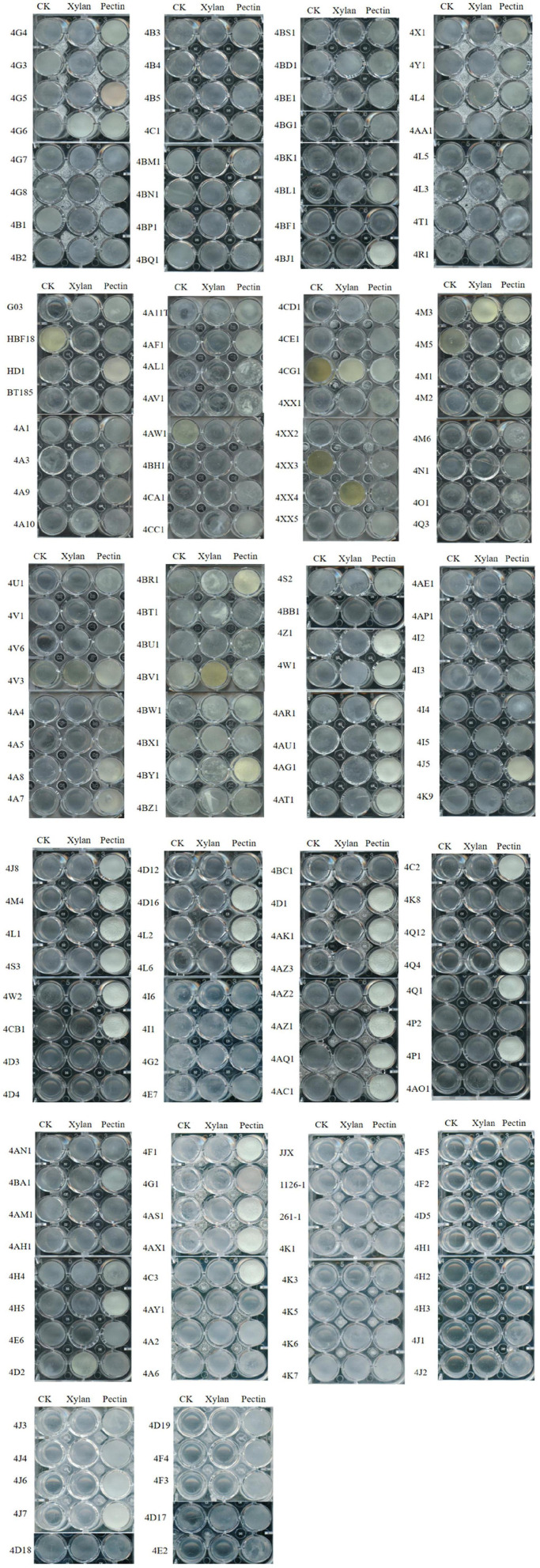
Effects of plant polysaccharides xylan and pectin on the biofilms formed by 170 Bt strains.

Combining with the scanned images for qualitative analysis of biofilms induced by pectin, there were 88 strains with strong biofilm formation, 69 strains with weak biofilm formation, and 13 strains without biofilm formation (Figure 1). When xylan was added, 13 Bt strains formed strong biofilms, 98 strains formed weak biofilms, and 59 strains did not form biofilms (Figure 1). This indicated that plant polysaccharides pectin and xylan can induce Bt strains to produce biofilms, and pectin can induce 92.4% of the tested Bt strains to form biofilms. Compared with xylan, pectin had a much better ability to induce Bt biofilm formation. Therefore, pectin was chosen for subsequent experiments.

### The Ability of Pectin to Induce Biofilm Formation Was Not Related to Bt Serotypes

**FIGURE 2:**
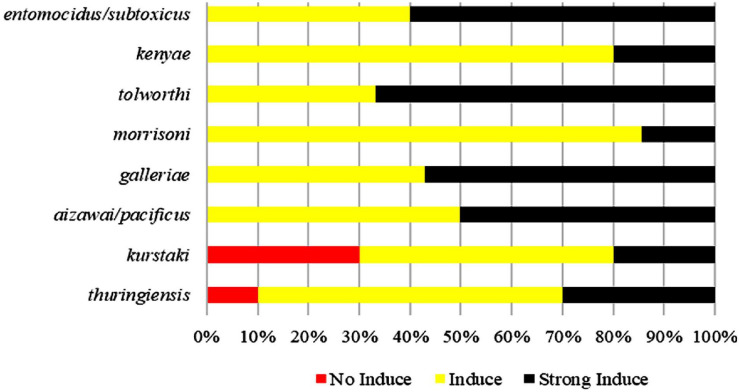
Comparison of induction ability of pectin on the biofilms formed by different Bt serotypes.

Pectin can induce all tested strains from the serotype subspecies *aizawai*/*pacificus*, *galleriae*, *morrisoni*, *tolworthi*, and *kenyae* of Bt to form biofilms (Table 1). Pectin can also induce the biofilm formation of most Bt strains from the serotypes *kurstaki* and *thuringiensis*. This indicated that the Bt biofilm induction by pectin was related to the genetic characteristics of the Bt strains, but not the serotype subspecies (Figure 2).

**TABLE 1:** Comparison of induction ability of pectin on the biofilms formed by different Bt serotypes.

**Bt sub.**	**Bt serotypes**	**BGSC no.**	**The induction capacity of pectin**		
			**Strong**	**Weak**	**No**
*Thuringiensis*	Serotype 1—serovar. *thuringiensis*	4A2	+		
	Serotype 1—serovar. *thuringiensis*	4A5	+		
	Serotype 1—serovar. *thuringiensis*	4A9		+	
	Serotype 1—serovar. *thuringiensis*	4A10		+	
	Serotype 1—serovar. *thuringiensis*	4A8		+	
	Serotype 1—serovar. *thuringiensis*	4A6		+	
	Serotype 1—serovar. *thuringiensis*	4A7	+		
	Serotype 1—serovar. *thuringiensis*	4A3		+	
	Serotype 1—serovar. *thuringiensis*	4A4		+	
	Serotype 1—serovar. *thuringiensis*	4A1			+
*Kurstaki*	Serotype 3a, 3b, 3c—serovar. *kurstaki*	4D2		+	
	Serotype 3a, 3b, 3c—serovar. *kurstaki*	4D1	+		
	Serotype 3a, 3b, 3c—serovar. *kurstaki*	4D16	+		
	Serotype 3a, 3b, 3c—serovar. *kurstaki*	4D17		+	
	Serotype 3a, 3b, 3c—serovar. *kurstaki*	4D18		+	
	Serotype 3a, 3b, 3c—serovar. *kurstaki*	4D19		+	
	Serotype 3a, 3b, 3c—serovar. *kurstaki*	4D5		+	
	Serotype 3a, 3b, 3c—serovar. *kurstaki*	4D12			+
	Serotype 3a, 3b, 3c—serovar. *kurstaki*	4D4			+
	Serotype 3a, 3b, 3c—serovar. *kurstaki*	4D3			+
*Aizawai/pacificus*	Serotype 7—serovar. *aizawai/pacificus*	4J5	+		
	Serotype 7—serovar. *aizawai/pacificus*	4J8		+	
	Serotype 7—serovar. *aizawai/pacificus*	4J7	+		
	Serotype 7—serovar. *aizawai/pacificus*	4J6	+		
	Serotype 7—serovar. *aizawai/pacificus*	4J4	+		
	Serotype 7—serovar. *aizawai/pacificus*	4J2		+	
	Serotype 7—serovar. *aizawai/pacificus*	4J3		+	
	Serotype 7—serovar. *aizawai/pacificus*	4J1		+	
*Galleriae*	Serotype 5a, 5b—serovar. *galleriae*	4G6	+		
	Serotype 5a, 5b—serovar. *galleriae*	4G5	+		
	Serotype 5a, 5b—serovar. *galleriae*	4G1	+		
	Serotype 5a, 5b—serovar. *galleriae*	4G3	+		
	Serotype 5a, 5b—serovar. *galleriae*	4G7		+	
	Serotype 5a, 5b—serovar. *galleriae*	4G4		+	
	Serotype 5a, 5b—serovar. *galleriae*	4G2		+	
*Morrisoni*	Serotype 8a, 8b—serovar. *morrisoni*	4K9	+		
	Serotype 8a, 8b—serovar. *morrisoni*	4AA1		+	
	Serotype 8a, 8b—serovar. *morrisoni*	4K8		+	
	Serotype 8a, 8b—serovar. *morrisoni*	4K7		+	
	Serotype 8a, 8b—serovar. *morrisoni*	4K5		+	
	Serotype 8a, 8b—serovar. *morrisoni*	4K3		+	
	Serotype 8a, 8b—serovar. *morrisoni*	4K1		+	
*Tolworthi*	Serotype 9—serovar. *tolworthi*	4L1	+		
	Serotype 9—serovar. *tolworthi*	4L2	+		
	Serotype 9—serovar. *tolworthi*	4L4	+		
	Serotype 9—serovar. *tolworthi*	4L5		+	
	Serotype 9—serovar. *tolworthi*	4L3		+	
	Serotype 9—serovar. *tolworthi*	4L6	+		
*Kenyae*	Serotype 4a, 4c—serovar. *kenyae*	4F1	+		
	Serotype 4a, 4c—serovar. *kenyae*	4F5		+	
	Serotype 4a, 4c—serovar. *kenyae*	4F2		+	
	Serotype 4a, 4c—serovar. *kenyae*	4F3		+	
	Serotype 4a, 4c—serovar. *kenyae*	4F4		+	
*Entomocidus/subtoxicus*	Serotype 6—serovar. *entomocidus/subtoxicus*	4I5	+		
	Serotype 6—serovar. *entomocidus/subtoxicus*	4I3	+		
	Serotype 6—serovar. *entomocidus/subtoxicus*	4I2	+		
	Serotype 6—serovar. *entomocidus/subtoxicus*	4I4		+	
	Serotype 6—serovar. *entomocidus/subtoxicus*	4I1		+	

*BGSC, Bacillus Genetic Stock Center.*

### Pectin Promoted the Biofilms Formed by Bt Cells in the Logarithmic Growth Phase at the Air–Liquid Interface

**FIGURE 3:**
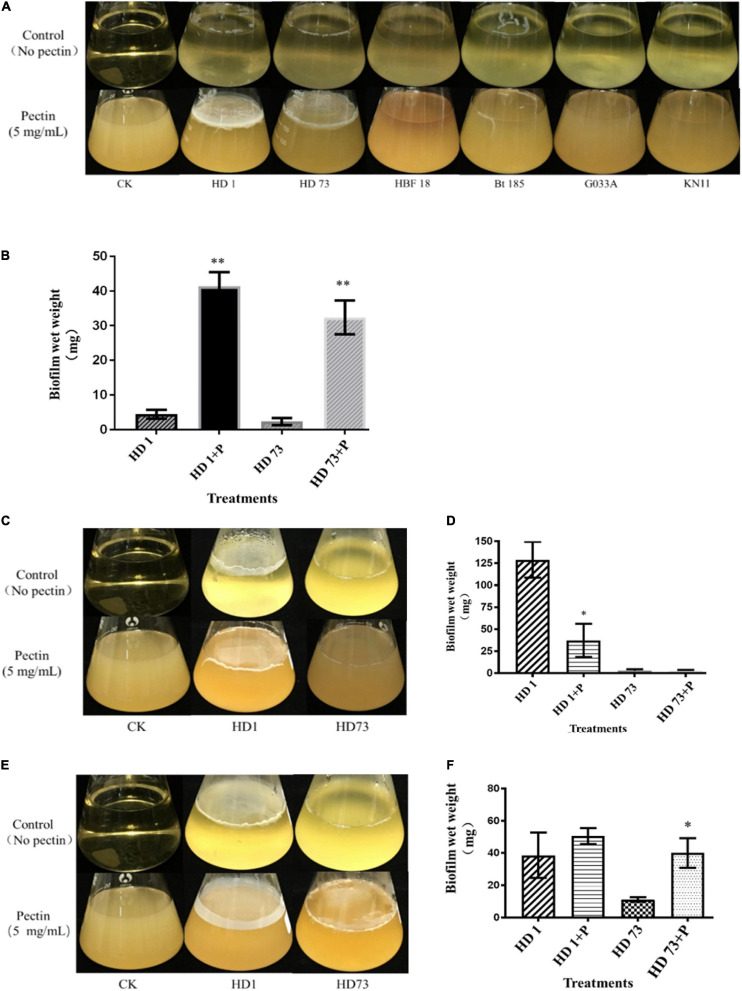
Effects of pectin on the biofilms formed by industrial Bt cellsat the air–liquid interfaces. **(A)** Bt strains HD1, HD73, Bt185, HBF18, Bt185, G033A, and KN11 that are currently used for industrial production were selected to test the effect of pectin on the biofilm formation of Bt logarithmic growth phase cells. **(B)** Quantification of the results of Bt HD1 and HD73 from **(A)**. **(C)** Bt strains HD1 and HD73 that are currently used for industrial production were selected to test the effect of pectin on the biofilm formation of Bt sporangial phase cells. **(D)** Quantification of the results of Bt HD1 and HD73 from **(C)**. **(E)** Bt strains HD1 and HD73 that are currently used for industrial production were selected to test the effect of pectin on the biofilm formation of Bt lysis phase cells. **(F)** Quantification of the results of Bt HD1 and HD73 from **(E)**. P in the figures B–F represents pectin. * and ** represent a significant difference and an extremely significant difference between the biofilms formed by the Bt strains without or with pectin induction, respectively.

Bt strains HD1, HD73, HBF18, Bt 185, G033A, and KN11 with industrial production applications from the above strains were selected to test the abilities to form biofilms at the air–liquid interface. It turned out that only strains HD1, HD73, and Bt185 were able to form weak circular biofilms without pectin. When the pectin was added, Bt strains HD1 and HD73 were significantly enhanced. That is, they can form intact biofilms with severely wrinkled surface at the air–liquid interface, and the thickness was greater than 1 mm (Figure 3A). Therefore, we continued to determine the effects of pectin on the growth and motility capacity of HD1 and HD73, as well as the biofilm formed by the two Bt cells at different growth phases. The results further proved that the ability of pectin to induce Bt biofilm had individual strain differences.

Pectin-induced Bt biofilms formed at the air–liquid interface of 200 mL LB liquid medium were harvested, dried, and quantified (Figure 3B). The results showed that pectin increased the yield of Bt HD1 biofilm by 9.27 times, from 4.46 ± 1.26 mg to 41.34 ± 4.10 mg (*P* < 0.01), whereas that of Bt HD73 increased 13.66-fold by shifting from 2.37 ± 1.03 to 32.38 ± 4.84 mg (*P* < 0.01).

### Pectin Inhibited the Biofilms Formed by Bt Cells in the Sporangial Phase at the Air–Liquid Interface

Without pectin, Bt HD1 cells in the sporangial phase were able to form an intact biofilm at the gas–liquid interface, whereas Bt HD73 can only form a circular biofilm at the same condition. With the induction of pectin, Bt HD1 biofilm appeared as a thin film, whereas Bt HD73 biofilm formation did not change (Figure 3C). By adding pectin, the amount of biofilm formed by Bt HD1 cells in the sporangial phase was reduced from 128.99 ± 20.34 mg to 37.28 ± 18.89 mg (*P* > 0.05). And the yield of the biofilm formed by the sporangial phase of Bt HD73 was changed from 2.90 ± 1.64 mg and 2.67 ± 1.02 mg (*P* > 0.05) (Figure 3D). The results showed that pectin has an inhibitory effect on the biofilm formed by the sporangial cells of Bt HD1.

### Pectin Enhanced the Biofilms Formed by Bt Cells in the Lysis Phase at the Air–Liquid Interface

The cells of Bt HD1 and HD73 in the lysis phase (spore–ICP mixture) formed circular biofilms at the contact position of the flask at the gas–liquid interface during static culture. Under the induction of pectin, the cells in the lysis phase of Bt HD1 also formed a film-like biofilm at the gas–liquid interface, whereas the cells in the lysis phase of Bt HD73 cannot form an intact biofilm at the gas–liquid interface (Figure 3E). With the addition of pectin, both of Bt HD1 and HD73 formed better biofilms, in that the yields of biofilms changed from 38.64 ± 14.06 mg to 50.54 ± 5.00 mg for HD1 (*P* > 0.05), and from 11.18 ± 1.39 mg to 40.08 ± 9.21 mg for HD73 (*P* > 0.05). The biofilm yield of HD73 increased 3.58 times (Figure 3F). Herein, there were certain differences in the ability of pectin to induce biofilms formed by Bt cells in the lysis phase.

### The Dosage of Pectin Was Positively Correlated With the Yield of Biofilms Formed by Bt Cells in the Logarithmic Growth Phase or Lysis Phase at the Solid–Liquid Interfaces

**FIGURE 4:**
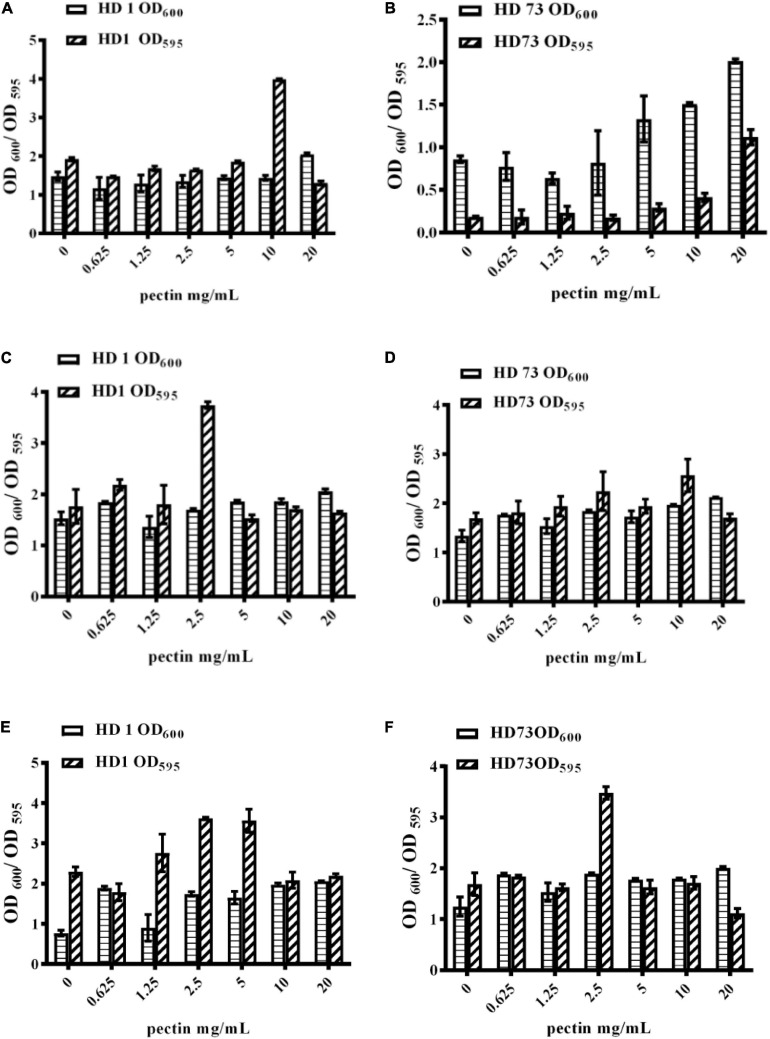
Effects of pectin dosage on the biofilms formed by Bt cells in different growth phases at the solid–liquid interfaces. **(A,B)** The logarithmic growth phase cells of Bt HD1 and HD73, respectively. **(C,D)** The sporangial phase cells of Bt HD1 and HD73, respectively. **(E,F)** The lysis phase cells of Bt HD1 and HD73, respectively.

With the increase of the pectin dosage, Bt HD1 cells from the logarithmic growth phase did not dramatically alter their growth, but it formed the strongest biofilm with the induction of 10 mg/mL pectin (Figure 4A). The OD_600_ values and biofilm formation of Bt HD73 cells from the logarithmic growth phase increased with the increase of pectin dosage, and 20 mg/mL pectin can induce the strongest biofilm (Figure 4B). Therefore, pectin can increase the biofilm formation of Bt cells in the logarithmic growth phase of strains HD1 and HD73.

With the increase of pectin dosage, the OD_600_ values of Bt HD1 and HD73 cells from the sporangial phase did not change significantly. Bt HD1 and HD73 reached the maximum biofilm formation by adding 2.5 and 10 mg/mL pectin, respectively (Figures 4C,D).

With the increase of the pectin dosage, the growth of Bt HD1 and HD73 in the lysis phase increased at most of the pectin dosages with an exception of 1.25 mg/mL for Bt HD1. The biofilm formation of HD1 cells from the lysis phase reached higher yields with the induction of 1.25, 2.5, or 5 mg/mL pectin, whereas pectin only promoted the biofilm formation of Bt HD73 cells from the lysis phase at 2.5 mg/mL (Figures 4E,F).

### Pectin Did Not Dramatically Change the Free-Living Growth of Bt HD1 and HD73

**FIGURE 5:**
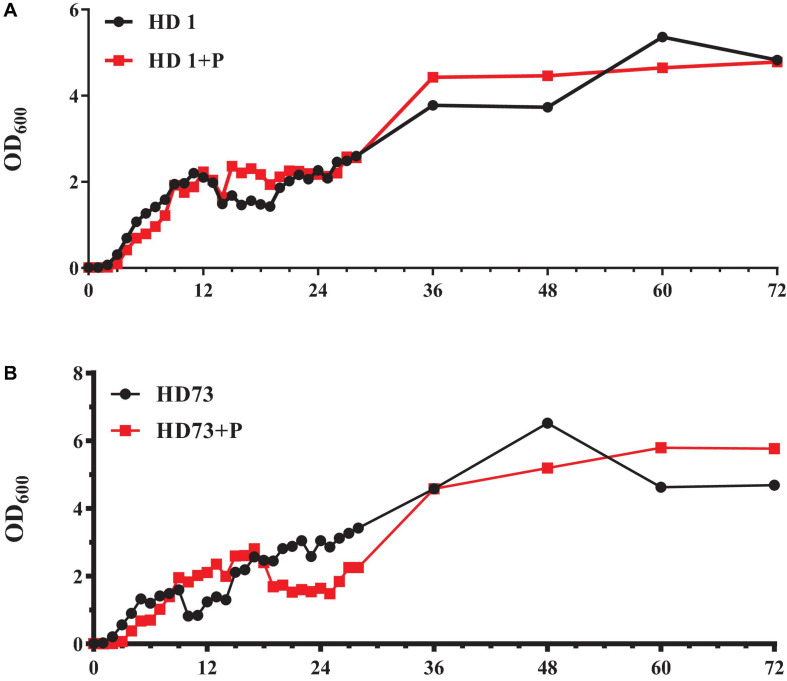
Growth curves of free-living Bt cells with or without the induction of pectin. **(A)** Bt HD1. **(B)** Bt HD73.

The number of Bt HD1 free-living cells induced by pectin exceeded that of Bt cells without pectin after culture for 12–23 and 28–48 h (Figure 5A). At most of the growth phases, the free-living growth of Bt HD73 was not promoted by pectin (Figure 5B).

### Pectin Did Not Affect the Cell Motility of Bt Strains

**FIGURE 6:**
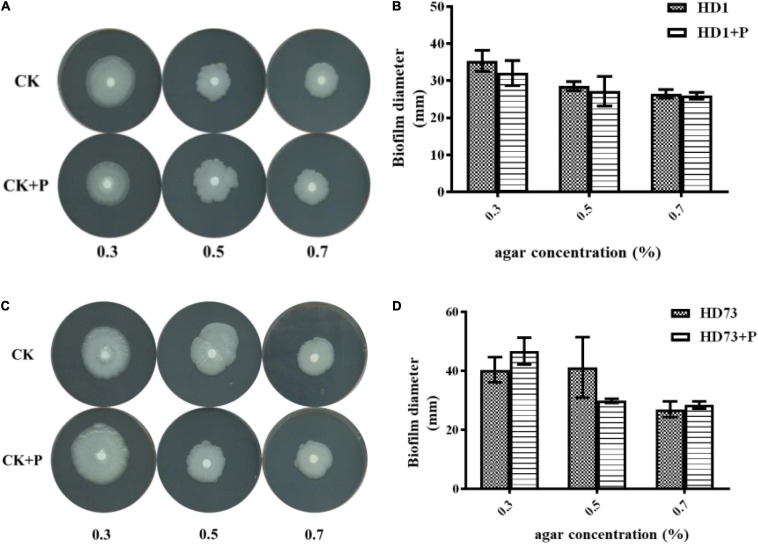
Effects of pectin on the cell motility of Bt strains HD1 and HD73. **(A,B)** HD1. **(C,D)** HD73.

In order to explore the effect of pectin on the cell motility of Bt strains, their swimming abilities and swarming abilities were measured on 90-mm semisolid plates with 0.3, 0.5, or 0.7% agar. The results showed that pectin did not affect the cell motility of Bt HD1 and HD73 (Figure 6).

### Pectin Can Improve the Biocontrol Activity of the Spore–ICP Mixture of Bt

**FIGURE 7:**
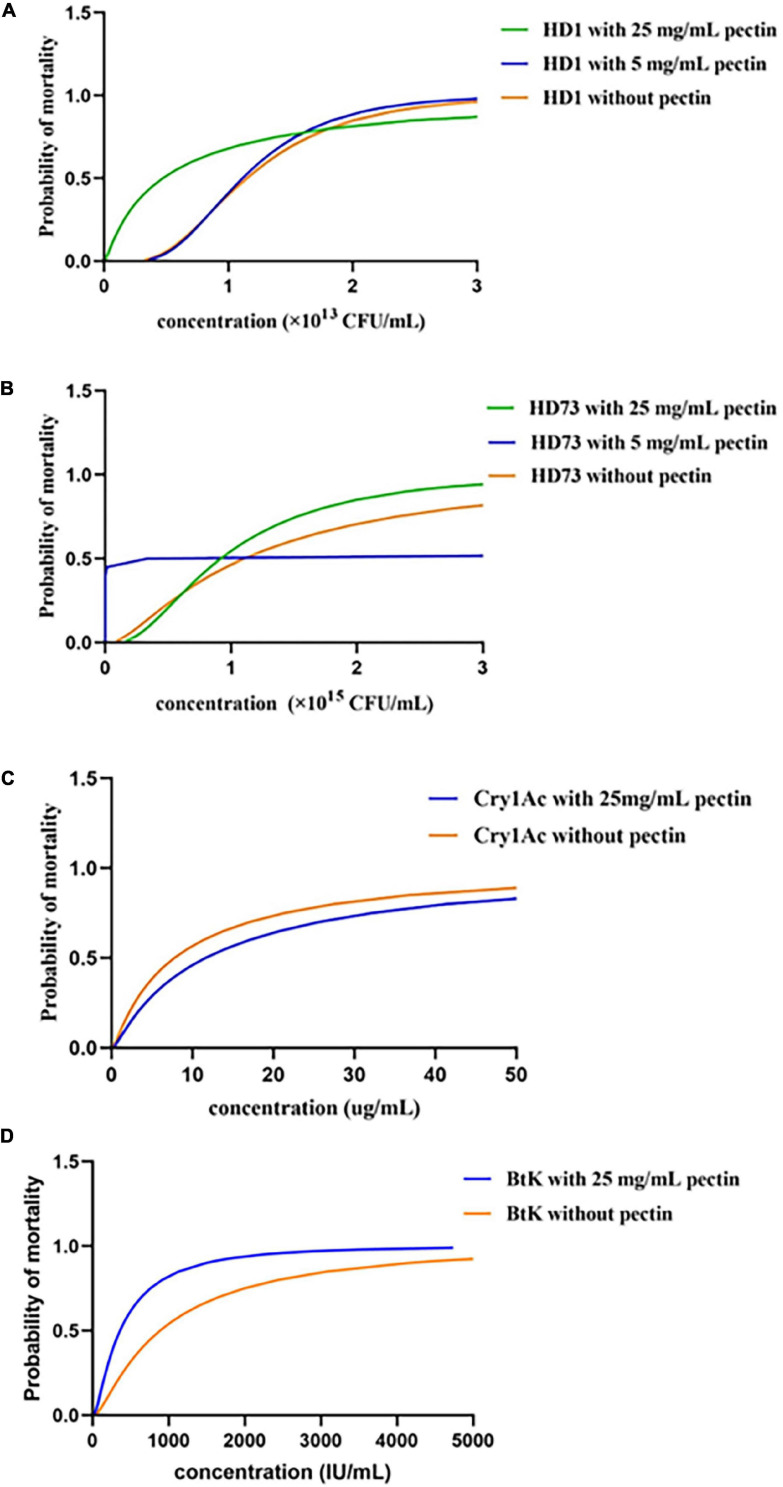
Effects of pectin on the biocontrol activities of Bt strains and preparations. **(A,B)** Effect of pectin on the biocontrol activities of the spore–ICP mixtures of Bt strains HD1 and HD73 against *P. xylostella*, respectively. **(C)** Effect of pectin on the biocontrol activity of Cry1Ac extracted from Bt HD73 against *P. xylostella*; the value of LC_50_ of Cry1Ac from HD73. **(D)** Effect of pectin on the biocontrol activity of BtK preparations against *P. xylostella.*

Pectin was explored for its effect on the biocontrol activity of Bt. The results showed that 25 mg/mL pectin significantly increased the mortalities of the spore–ICP mixtures of Bt HD1 (0.85 × 10^13^ CFU/mL) or HD73 (1.325 × 10^15^ and 2.65 × 10^15^ CFU/mL) against *P. xylostella* (Figures 7A,B). However, 5 mg/mL pectin had negative or no effect on the biocontrol activity of the strains against *P. xylostella* (Figures 7A,B), indicating that the promotion of pectin on biocontrol activity of the spore–ICP mixture of Bt HD1 was dosage-dependent. Pectin 25 mg/mL was used for subsequent experiments. The LC_50_ of HD1 for the spore–ICP mixture with 25 mg/mL pectin was 0.469 × 10^13^ CFU/mL. As a result, the HD1 treated with 25 mg/mL pectin was more toxic than HD1 strain without pectin. And the LC_50_ of HD73 with 5 mg/mL pectin was 0.332 × 10^15^ CFU/mL; it turned out that HD73 treated with 5 mg/mL pectin is more toxic than the spore–ICPs without pectin (Table 2).

**TABLE 2:** Dose–response insecticidal activities of the spore–ICPs of HD1 and HD73 strains.

**Strain**	**50% Lethal concentration (CFU/mL)**	***P***	**Slope**	χ^2^
	**(95% Confidence interval)**			
HD1	1.148 (0.886–1.442)	0.141	4.267	6.9
HD1 induced with 5 mg/mL pectin	1.111 (0.999–1.226)	0.306	4.779	4.818
HD1 induced with 25 mg/mL pectin	0.469	0.038	1.436	10.127
HD73	1.103 (0.469–1.426)	0.875	2.108	1.219
HD73 induced with 5 mg/mL pectin	0.332	0.222	0.093	5.705
HD73 induced with 25 mg/mL pectin	0.917 (0.478–1.170)	0.010	2.535	13.255

### Pectin Did Not Affect the Biological Activity of Cry1Ac Protein

Our data showed that pectin can improve the biocontrol activity of the spore–ICP mixture of Bt. In order to clarify whether this activity promotion was due to the effect of pectin on ICPs, the effect of pectin on the biocontrol activity of Cry1Ac protein was investigated by extraction of Cry1Ac from Bt HD73 with one ICP (Cry1Ac). The results showed that 25 mg/mL pectin can inhibit the toxicity of 33.33 μg/mL Cry1Ac against *P. xylostella*, but it had no effect on the toxicities of Cry1Ac at 100, 11.11, 3.07, and 1.234 μg/mL (Figure 7C), indicating that pectin did not promote the insecticidal activity of the Cry1Ac protein alone, and it may promote the production of other virulence factors in Bt. The LC_50_ of the Cry1Ac extracted from HD73 strains was 7.738 μg/mL without pectin. And with 25 mg/mL pectin, the LC_50_ was 11.583 μg/mL (Table 3).

**TABLE 3:** Dose–response insecticidal activity of the Cry1Ac of HD73 strains.

**Strain**	**50% Lethal concentration (μg/mL)**	***P***	**Slope**	**χ^2^**
	**(95% Confidence interval)**			
HD73	7.738 (5.609–10.631)	0.034	1.533	23.669
HD73 induced with 25 mg/mL pectin	11.583 (8.092–16.819)	0.004	1.524	30.25

### Pectin Improved the Biocontrol Activity of BtK Commercial Insecticides

Pectin 25 mg/mL can increase the mortality of 1.6 × 10^3^ and 1.6 × 10^2^ IU/mL BtK preparation against *P. xylostella* (Figure 7D). At 1.6 × 10^3^ and 1.6 × 10^2^ IU/mL, BtK preparation caused higher mortality in *P. xylostella.* The LC_50_ value for BtK preparation against *P. xylostella* was 884.197 IU/mL. And with the induction of 25 mg/mL pectin, the LC_50_ value for BtK preparation against *P. xylostella* was 358.781 IU/mL (Table 4).

**TABLE 4:** Dose/response insecticidal activity of BtK preparation.

**Strain**	**50% Lethal concentration (IU/mL)**	***P***	**Slope**	**χ^2^**
	**(95% Confidence interval)**			
BtK	884.197 (677.517–1180.735)	0.63	1.913	2.583
BtK induced with 25 mg/mL pectin	358.781 (211.212–592.811)	0.136	2.076	6.999

### Pectin Can Promote the Biocontrol Activities of Biofilms Formed by the Logarithmic Growth Phase or Lysis Phase of Bt Cells

**FIGURE 8:**
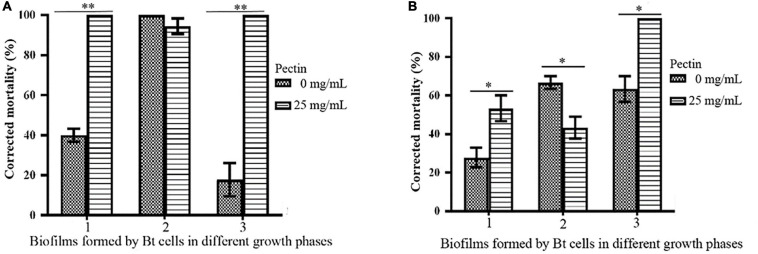
Effects of pectin on the toxicities of biofilms formed by Bt cells in different growth phases. **(A)** HD1. **(B)** HD73. 1, 2, 3: Biofilms formed by Bt cells in the logarithmic growth phase, the sporangial phase, and the lysis phase, respectively. * and ** represent a significant difference and an extremely significant difference between the biofilms formed by the Bt strains without or with pectin induction, respectively.

By inducing with 25 mg/mL pectin, the toxicities of biofilms formed by the logarithmic growth phase or lysis phase of Bt HD1 and HD73 against *P. xylostella* were significantly higher than the controls. However, pectin inhibited the insecticidal activity of the biofilm formed by Bt HD73 in the sporangial phase against *P. xylostella* (Figure 8).

## Discussion

Although bacterial biofilms are harmful to human society in many cases, people have gradually realized the beneficial effects of bacterial biofilms in agriculture and industry (Bogino et al., 2013; Berlanga and Guerrero, 2016). They can be used for plant protection, bioremediation, wastewater treatment, and corrosion prevention (Morikawa, 2006; Singh et al., 2006, 2019; Edwards and Kjellerup, 2013; Naidoo and Olaniran, 2013). For example, biofilms of rhizosphere bacteria such as *B. subtilis*, *Pseudomonas*, *Streptomyces*, and *Serratia marcescens* can be used as biological control agents (Bais et al., 2004; Vejan et al., 2016; Arrebola et al., 2019). Bacterial biofilms such as *Aspergillus* and *Enterobacter* can be used as biological fertilizers (Bais et al., 2004; Khan et al., 2018; Singhalage et al., 2019). Compared with the researches of harmful bacterial biofilms, beneficial bacterial biofilms are still in their infant.

Plant polysaccharides are important biological macromolecules in plants, as well as one of the four basic substances that maintain and ensure normal biological activities. They had biological activities, including regulating immunity, antiradiation, antibacteria, and antivirus (Chen et al., 2016). Plant polysaccharides can induce the formation of biofilms by serving as an initial carbon source during the formation of biofilms or being degraded into galactose to be bound to the extracellular matrix (Beauregard et al., 2013). Plant polysaccharides are signal factors for the formation of *B. subtilis* biofilm (Beauregard et al., 2013). Pectin is a complex plant polysaccharide, constituting of the main component of the plant cell wall. It can be used as a carbon source to promote the formation of the extracellular matrix of strains and induce the formation of biofilm as environmental cues, whereas xylan is a polymer of xylose, a pentose. It is present mostly in fiber cells, contrary to pectin. However, the relationship between plant polysaccharides and the beneficial biofilms formed by microbial pesticide Bt, as well as its insecticidal activity, is still an unsolved mystery. In this study, a 24-well plate method was used to determine the polysaccharide-induced biofilms formed by 170 Bt strains, combining with the scanned images for qualitative analysis of the biofilms. The results showed that pectin can induce much better biofilms formed by Bt than that of xylan. Hence, we chose pectin to study its modulation on biofilm formation and insecticidal activities of Bt strains. And there was no difference in the abilities of pectin and xylan to induce biofilms among Bt serotypes. We further selected six Bt strains with industrial production applications to test their abilities to form biofilms at the air–liquid interface. It turned out that only strains HD1, HD73, and Bt185 can form weak biofilms without pectin. When the pectin was added, biofilm formation of Bt strains HD1 and HD73 was significantly enhanced. Therefore, we continued to determine the effects of pectin on the growth and motility capacity of HD1 and HD73, as well as the biofilm formed by the two Bt cells at different growth phases. In PubMed, there were 2,915 articles related to Bt strains searched using the keyword “*thuringiensis* strains,” among which there were 200 articles about HD1 strain (6.9%) and 117 articles about HD73 strain (4.0%). Both strains accounted for more than 10%. According to statistics, there were more than thousands of Bt strains stocked in the world. Therefore, the selection of HD1 and HD73 strains as the key research objects was widely representative for 170 tested Bt strains.

The biofilms formed under high nutrient conditions were denser than low nutrient levels (Sutherland, 2001). Our results showed that Bt biofilms first formed ring-shaped bacterial aggregates at the gas–liquid interface. After culturing under pectin induction for 48 h, intact membrane biofilms of HD1 and HD73 can be observed at the air–liquid interface. After that, the structure of the biofilm became more complex, showing increased thickness and severe surface wrinkles. Bt had three growth phases (logarithmic growth phase, sporangial phase, and lysis phase) in planktonic state and the field. Hence, the effects of pectin on the biofilms formed by Bt cells at the three different growth phases were investigated. Pectin had differences in the biofilm formation of Bt cells in different growth phases. At the air–liquid interface, pectin induced Bt HD1 and HD73 cells in the logarithmic growth phase and Bt HD73 cells in the lysis phase to form stronger biofilms, but it inhibited Bt HD1 cells in the sporangial phase to form better biofilm.

Bacterial motility is a key factor in the formation of biofilms. Under static conditions, bacteria first reach a position suitable for biofilm formation and then begin the development of biofilm (Houry et al., 2010). As the biofilm ages, the transcription of flagellin continues to decline, and the bacterial population in the biofilm, including part of the motile cells at the edge of the colony, is heterogeneous (Houry et al., 2012). The increase in biofilm biomass when pectin was used to treat *Bacillus amyloliquefaciens* was mainly due to the production of surfactant and iturin (Wu et al., 2015). When pectin was used to induce biofilms formed by Bt cells in the growth phase, the biofilm production was also increased, but it did not affect the cell motility of Bt subsp. *kurstaki* HD1 and HD73. It is of great interest to elucidate what special substances contribute to this phenomenon shortly.

Pectin strongly induced the biofilm formation of Bt cells in the logarithmic growth phase, but exhibited different effects on Bt cells in the sporangial phase and lysis phase. This may be because spore differentiation and biofilm formation belong to different but related regulatory pathways. In Bt biofilm, the gene expressions of the four cell types—toxicity, necrosis, toxicity/necrosis, and necrosis/sporulation—were highly different (Verplaetse et al., 2015). The spore differentiation of Bt was mainly regulated by the phosphorylation pathway of Spo0A-P, whereas the pathways of Bt biofilm formation needs further studies (Verplaetse et al., 2015; Xu et al., 2017).

Pectin-rich amendment enhances soybean growth promotion and modulation mediated by *Bacillus velezensis* (Hassan et al., 2019). In this study, pectin can promote the biocontrol activities of biofilms formed by the logarithmic growth phase or lysis phase of Bt cells. It also found that 25 mg/mL pectin can improve the biocontrol activity of the spore–ICP mixtures of Bt HD1 and HD73, but the low one cannot promote the insecticidal activity. We speculated that pectin may provide rich nutrition as a carbon source to promote the biofilm formation and to improve the insecticidal activities. The reason why 25 mg/mL pectin cannot promote the insecticidal activity of the Cry1Ac extracted from Bt HD73 is that no living bacteria existed at this time and no production of biofilm. Thus, the insecticidal activity of Cry1Ac cannot be improved by pectin induction. When the BtK preparation was 1.6 × 10^3^ and 1.6 × 10^2^ IU/mL, 25 mg/mL pectin can increase its mortality of *P. xylostella*. And the LC_50_ values were 358.781 and 884.197 IU/mL with or without pectin, respectively. Herein, plant polysaccharides can promote the biocontrol activity of Bt against insect pests.

In summary, biological pesticide Bt can form biofilms on the surface of vegetable leaves rich in polysaccharides. The lifestyle of bacteria in the biofilm state is completely different from the planktonic lifestyle. Therefore, how to apply biofilm research finding to biological control of insect pests with Bt in the field has become the key concern of Bt colleagues. Main Bt formulations were wettable powders. When used in the field, they are dissolved in water and sprayed. Therefore, we directly add pectin and other plant polysaccharides to commercially produced Bt for biofilm and insecticidal activity determination, which was more conducive to the conversion of scientific research results into productivity. This study primarily elucidated the effects of plant polysaccharides on biofilm formation and insecticidal activities of Bt. Our findings confirmed that plant polysaccharides modulate biofilm formation and insecticidal activities of Bt. Indeed, the capacity of Bt to form biofilms when in contact with plant polysaccharides could be an advantage and thus build a foundation for the construction of biofilm-type Bt biopesticides with long-term field duration. Future research can be carried out in the mode of actions of pectin to further broaden the depth and breadth of bacterial biofilms.

## Data Availability Statement

The original contributions presented in the study are included in the article/supplementary material, further inquiries can be directed to the corresponding author/s.

## Author Contributions

TH conceived the research direction of this manuscript. ML and CS performed the experiments. ML, CS, XL, and YY analyzed the experimental results. ML and WK wrote the manuscript. XG, TH, and CS reviewed the manuscript. All authors contributed to the article and approved the submitted version.

## Conflict of Interest

The authors declare that the research was conducted in the absence of any commercial or financial relationships that could be construed as a potential conflict of interest.

## Footnotes

1 http://www.chinapesticide.org.cn/hydt/4302.jhtml
